# The nematode α-catenin ortholog, HMP1, has an extended α-helix when bound to actin filaments

**DOI:** 10.1016/j.jbc.2022.102817

**Published:** 2022-12-17

**Authors:** Erumbi S. Rangarajan, Emmanuel W. Smith, Tina Izard

**Affiliations:** 1 Cell Adhesion Laboratory, UF Scripps, Jupiter, Florida, USA; 2 The Skaggs Graduate School, The Scripps Research Institute, Jupiter, Florida, USA

**Keywords:** actin, cancer, cell adhesion, cell junction, cell migration, cell signaling, cryoEM, cryogenic electron microscopy, CTF, contrast transfer function, EDTA, ethylenediaminetetraacetic acid, FABD, F-actin binding domain, GST, glutathione S-transferase, JEOL, Japan Electron Optics Laboratory, SAXS, small-angle X-ray scattering, SEC, size exclusion chromatography

## Abstract

The regulation of cell–cell junctions during epidermal morphogenesis ensures tissue integrity, a process regulated by α-catenin. This cytoskeletal protein connects the cadherin complex to filamentous actin at cell–cell junctions. The cadherin–catenin complex plays key roles in cell physiology, organism development, and disease. While mutagenesis of *Caenorhabditis elegans* cadherin and catenin shows that these proteins are key for embryonic morphogenesis, we know surprisingly little about their structure and attachment to the cytoskeleton. In contrast to mammalian α-catenin that functions as a dimer or monomer, the α-catenin ortholog from *C. elegans*, HMP1 for humpback, is a monomer. Our cryogenic electron microscopy (cryoEM) structure of HMP1/α-catenin reveals that the amino- and carboxy-terminal domains of HMP1/α-catenin are disordered and not in contact with the remaining HMP1/α-catenin middle domain. Since the carboxy-terminal HMP1/α-catenin domain is the F-actin-binding domain (FABD), this interdomain constellation suggests that HMP1/α-catenin is constitutively active, which we confirm biochemically. Our perhaps most surprising finding, given the high sequence similarity between the mammalian and nematode proteins, is our cryoEM structure of HMP1/α-catenin bound to F-actin. Unlike the structure of mammalian α-catenin bound to F-actin, binding to F-actin seems to allosterically convert a loop region of the HMP1/α-catenin FABD to extend an HMP1/α-catenin FABD α-helix. We use cryoEM and bundling assays to show for the first time how the FABD of HMP1/α-catenin bundles actin in the absence of force. Collectively, our data advance our understanding of α-catenin regulation of cell–cell contacts and additionally aid our understanding of the evolution of multicellularity in metazoans.

Cell–cell contacts are essential in the maintenance of the architecture of tissues and the communication between cells. At the center of these cell–cell junctions is the cadherin receptor. Upon binding to calcium, the extracellular domain of one cadherin binds to that of a neighboring cell. In the cytosol, the transmembrane cadherin binds β-catenin, which binds the cytoskeletal protein α-catenin. The resulting cadherin-catenin complex is key to cell physiology, organism development, and disease (1, 2, 3, 4, 5, 6, 7, 8).

The evolution of the regulation of cell–cell adhesion by the cadherin–catenin complex coincided with the formation of specific body plans from epithelial sheets (9). The cadherin–catenin complex is conserved in metazoans and several pre-metazoans and unikonts. In the nematode *Caenorhabditis elegans*, proteins equivalent in structure and function to mammalian cadherin, β-catenin, and α-catenin are HMR1, HMP2, and HMP1, respectively (10, 11, 12, 13).

The connection to the actin cytoskeleton through α-catenin is essential for the morphogenetic epithelial process. One functional example of *C. elegans* is the ventral closure (12, 14). Mutations of the cadherin–catenin complex in the roundworm result in severe morphological defects during embryogenesis. Specifically, loss of HMP1/α-catenin leads to failure to establish ventral epidermal cell contacts resulting in an uncovered head, as seen for loss of HMR1/cadherin. Without HMP1/α-catenin, leader cells cannot be recruited to new cell–cell contacts. Further, HMP1/α-catenin mutants that are defective in binding to F-actin inhibit embryonic elongation. Thus, the anchoring of HMR1/cadherin to the actin cytoskeleton is essential during embryonic elongation (15). While a clear role of force has been established for mammalian α-catenin regulation (16, 17, 18, 19, 20, 21, 22), this process has not been thoroughly studied in *C. elegans*. Specifically, mammalian cells recruit vinculin when α-catenin is under force (23, 24). Binding of vinculin to α-catenin reinforces cell junctions. In contrast, roundworm epithelial cells do not express vinculin (25). The HMP1/α-catenin connection to the actin cytoskeleton presents is a major transmitter and transducer of actomyosin contractility to provide continuous tension across junctions. How the actomyosin tension on cell–cell junctions is distributed and controlled, and how it regulates morphological changes in the embryo remains to be seen (20, 26).

α-Catenin is a flexible all-helical protein comprised of an amino-terminal domain that is connected to its middle and amino-terminal F-actin binding domain (FABD) (27). The amino-terminal domain consists of two four-helix bundle domains that share one long central α-helix. The middle domain has a second set of two four-helix bundle domains, M1 and M2, that share one long central α-helix plus a third four-helix bundle, M3. The FABD is a five-helix bundle.

Despite over 39% sequence identity and 60% similarity along the lengths of human α-catenin and HMP1/α-catenin polypeptide chains, HMP1/α-catenin is a monomer (11), while mammalian α-catenin dimerizes in the cytosol (5). However, neuronal murine αN-catenin does not dimerize (28). If dimerization does occur, this is accomplished by two amino-terminal α-helices that dimerize to form a four-helix bundle. Further, deletion of the unstructured amino terminus stabilizes dimer formation (28).

Key known α-catenin structures are the complex between α-catenin and β-catenin (29), dimeric α-catenin (27), and, more recently, the FABD of α-catenin bound to filamentous actin (17, 18). Structural data for HMP1/α-catenin are limited to the middle domain (20, 30). Here, we provide structural insights into the unbound HMP1/α-catenin and the HMP1/α-catenin FABD bound to actin filaments. Our size exclusion multiangle light scattering data determine that HMP1/α-catenin is a monomer in solution. HMP1/α-catenin is a flexible protein consistent with lack of cryogenic electron microscopy (cryoEM) density for the amino-terminal and FABDs in our 4.8 Å structure. The HMP1/α-catenin FABD moves freely as a bead on a string, suggesting that HMP1/α-catenin is constitutively active, which we confirm by actin co-sedimentation assays. Surprisingly, our 3.4 Å HMP1/α-catenin bound to F-actin cryoEM structure suggests that F-actin binding causes a loop region of the HMP1/α-catenin FABD to extend an FABD α-helix. This extended α-helix increases the HMP1/α-catenin buried surface area compared to mammalian α-catenin and might be relevant in stabilizing the FABD helical bundle. We use cryoEM and bundling assays to show how the FABD of HMP1/α-catenin bundles actin in the absence of force. Collectively, our data advance our understanding of α-catenin regulation of cell–cell contacts also with respect to the evolution of multicellularity in metazoans.

## Results

### Distinct nematode and human α-catenin quaternary structures

**Figure 1:**
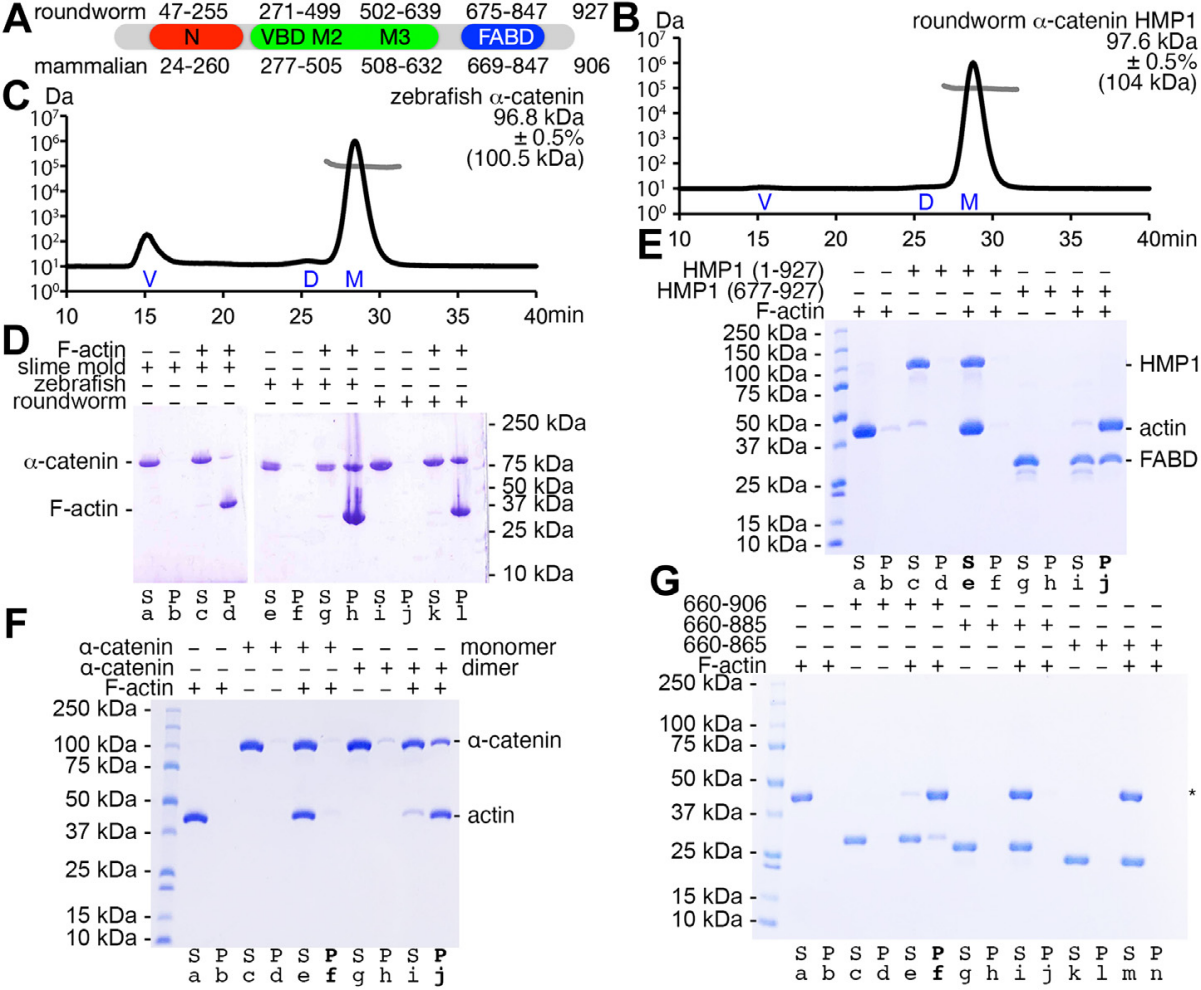
**HMP1/α-catenin is a monomer that binds F-actin.***A*, schematic domain structure of roundworm and mammalian α-catenin. N, amino-terminal domain (*red*); VBD, vinculin binding domain; M2 and M3, middle subdomains two and three; FABD, F-actin binding domain (*blue*). *B*, HMP1/α-catenin is a monodisperse monomer, while (*C*) zebrafish α-catenin shows some aggregation and dimerization as analyzed by size exclusion chromatography multiangle light scattering. The ordinate shows the experimental absolute mass (*light gray trace*) on a logarithmic scale without showing the second ordinate that represents the light scattering. The abscissa is the elution time for the light scattering profile (*black trace*). Values in parentheses are the calculated molecular weights of the corresponding polypeptide chains. V, void; D, dimer; M, monomer. *D*, monomeric HMP1/α-catenin co-sediments with F-actin (*lanes k and l*) and remains in solution without F-actin (*lanes i and j*). *Danio reiro* (zebrafish, *lanes e-h*) and *Dictyostelium discoideum* (slime mold, *lanes a-d*) α-catenins were used as controls. S, supernatant; P, pellet. *E*, full-length HMP1/α-catenin does not bundle F-actin (*lanes e and f*), while HMP1/α-catenin FABD (*lanes i and j*) exhibits bundling of F-actin. The F-actin (*lanes a and b*) and HMP1/α-catenin (*lanes c and d*) controls remain in solution. S, supernatant; P, pellet. *F*, the full-length human α-catenin monomer does not bundle F-actin (*lanes e and f*), while the dimer shows clear bundling (*lanes i and j*) of F-actin. Control F-actin (*lanes a and b*) remained in solution. S, supernatant; P, pellet. *G*, the human α-catenin FABD (residues 660–906) shows efficient bundling of F-actin (*lanes e and f*) while carboxy-terminal truncations did not show any apparent bundling (residues 660 to 885, *lanes i and j*; and 660 to 865, *lanes m and n*). Control F-actin (*lanes a and b*) remained in solution. S, supernatant; P, pellet; *asterisk*, actin.

The nematode *C. elegans* has an α-catenin homolog, named HMP1 for humpback. HMP1/α-catenin shares over 39% sequence identity throughout the human α-catenin polypeptide chain (Figs. 1*A* and S1, *A* and *B*). While vertebrate α-catenins are monomeric or dimeric depending on their location and function, the roundworm HMP1/α-catenin does not dimerize (11) (Fig. 1*B* and Table 1). We used the zebrafish (*Danio reiro*) α-catenin as a control that, in comparison, shows traces of possible dimerization as analyzed by size exclusion chromatography (SEC) and multiangle light scattering (Fig. 1*C*).

**Table 1:** Absolute molar mass determination by size exclusion chromatography multiangle light scattering (SEC-MALS)

	molar mass	Calculated molecular weight	Mass fraction	
(A) Monomeric *C. elegans* HMP1/α-catenin				
	97.6 kDa ± 0.5%	103.99 kDa	1.00	
(B) Monomeric *D. rerio* α-catenin				
	96.8 kDa ± 0.5%	101.2 kDa	1.00	

Individual peak fractions corresponding to HMP1/α-catenin or zebrafish α-catenin were pooled from previous size exclusion chromatography (SEC) and used for SEC-MALS analyses. The absolute molar mass was analyzed using Astra 6.0.

(A) The monomeric HMP1/α-catenin SEC pool remains a monomer on the SEC-MALS.

(B) The monomeric zebrafish α-catenin SEC pool shows some dimerization by SEC-MALS.

### HMP1/α-catenin co-sediments with F-actin

Loss of functions mutants of HMP1/α-catenin cause circumferential F-actin bundles to detach from the plasma membrane. This process results in the folding of the dorsal epidermis and the Humpback phenotype (12). HMP1/α-catenin was initially reported to not bind F-actin (11). More recently, phosphorylated HMP1/α-catenin bound to F-actin and the roundworm β-catenin ortholog HMP2 with low micromolar affinity (30, 31). However, we found that unphosphorylated HMP1/α-catenin bound F-actin (Fig. 1*D*). We used the zebrafish (*D. reiro*) and the slime mold (*Dictyostelium discoideum*) α-catenin as controls.

### The FABD of HMP1/α-catenin bundles F-actin

Truncations of the FABD of α-catenin affect its binding to F-actin (17). To put these data in the context of bundling F-actin, we generated the isolated human α-catenin FABD (residues 660–906) and carboxy-terminal truncations (residues 660–885 and 660–865) and the HMP1/α-catenin FABD (residues 677–927). Low speed sedimentation (10,000*g*) analyses allowed F-actin bundling readouts compared with high-speed sedimentation (100,000*g*) analyses. In the absence of actin binding proteins, F-actin remains in the supernatant under low-speed conditions while F-actin pellets upon high-speed centrifugation.

**Figure 2:**
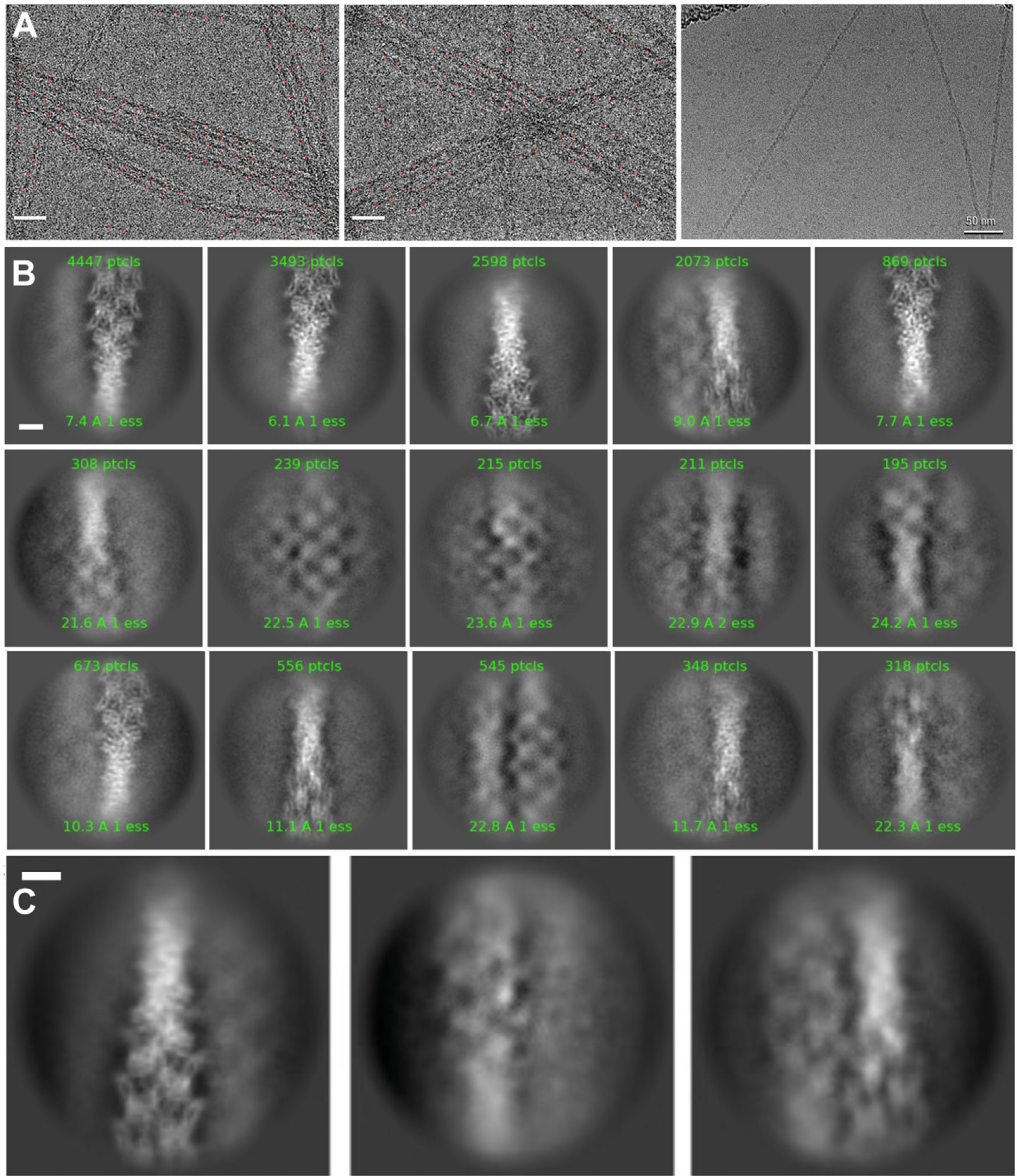
**Cryogenic electron microscopy micrograph images show actinbundling promoted by the addition of the HMP1/α-catenin F-actin binding domain (FABD).***A*, (*Left* and *middle*) representative micrographs show evidence of actin bundling by HMP1/α-catenin. *Red circles* indicate the picked particles that were used for 2D classification. (*Right*) Representative micrograph of F-actin alone (control). Scale bars, 50 nm. *B*, 2D classification with high degree of F-actin bundling of enriched 2D classes containing bundled actin filament particles. Scale bar, 5 nm. *C*, examples of 2D classes, where the FABD of HMP1/α-catenin seems to bridge two adjacent actin filaments. Scale bar, 5 nm.

We observed that the HMP1/α-catenin FABD bundled F-actin (Figs. 1*E* and 2*A*). Recent total internal reflection fluorescence microscopy showed that bundling by the mammalian α-catenin FABD was increased upon motor activation (18). Interestingly, we observed the HMP1/α-catenin FABD bridging two actin filaments in the absence of myosin (Fig. 2, *B* and *C*). However, we only observed F-actin bundling when we assayed with the FABD but not with full-length HMP1/α-catenin (Fig. 1*E*). Further, full-length monomeric human α-catenin did also not bundle F-actin while full-length dimeric human α-catenin bundled F-actin (Fig. 1*F*). However, when truncating the carboxy terminus on the human α-catenin FABD (Δ886–906 or Δ866–906) bundling is no longer observed compared to the human α-catenin FABD that was not truncated (residues 660–906) (Fig. 1*G*).

### Distinct nematode and mammalian α-catenin structures bound to F-actin

Although full-length HMP1/α-catenin binds F-actin (Fig. 1*D*), we only observed partial decoration of F-actin as determined by 2D classification. The partial decoration consistently led to 3D reconstructions that lacked density for HMP1/α-catenin. Extensive efforts to enrich the decoration through careful curation of 2D classes did also not lead to decorated HMP1/α-catenin actin filaments.

**Figure 3:**
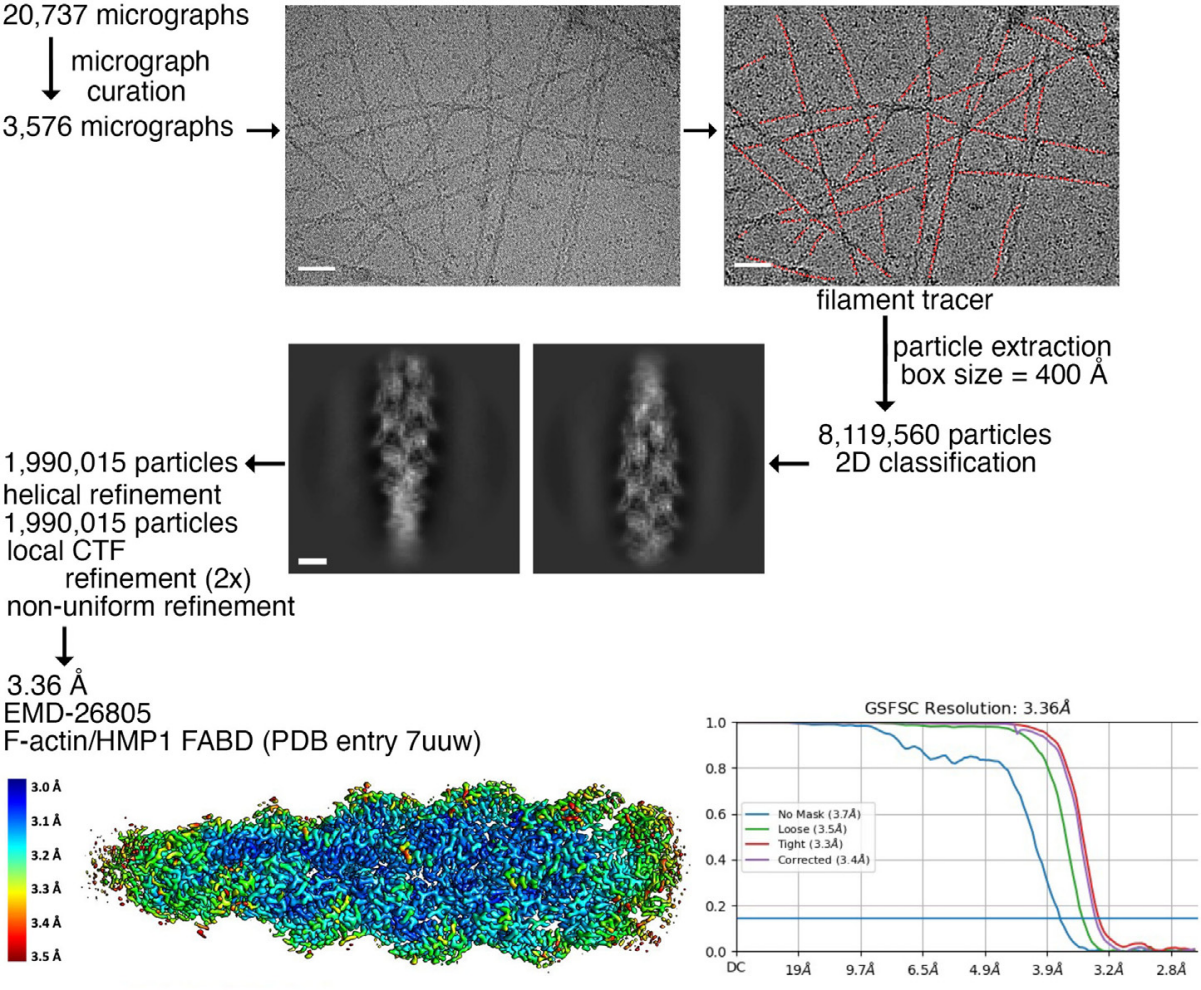
**Cryogenic electron microscopy data processing workflow for F-actin bound by the HMP1/α-catenin F-actin binding domain (FABD)**. Representative micrographs are shown before (*top* and *left*) and after particle picking (*top* and *right*; *red traces*). Representative 2D classes of decorated actin filaments are shown with a box size of 400 Å. The final 3D reconstruction workflow led to the reconstruction of a 3.36 Å resolution map, as estimated in cryoSPARC (48) using a Fourier shell correlation cut-off value of 0.143. The EMDB deposition identifier (EMD-28805) is indicated, and the fitted structure and corresponding PDB deposition identifier (7uuw) are also noted. The scale bars on the micrographs (*top*) correspond to 50 nm. The scale bar on the 2D class average (*middle*) corresponds to 5 nm. CTF, contrast transfer function.

**Figure 4:**
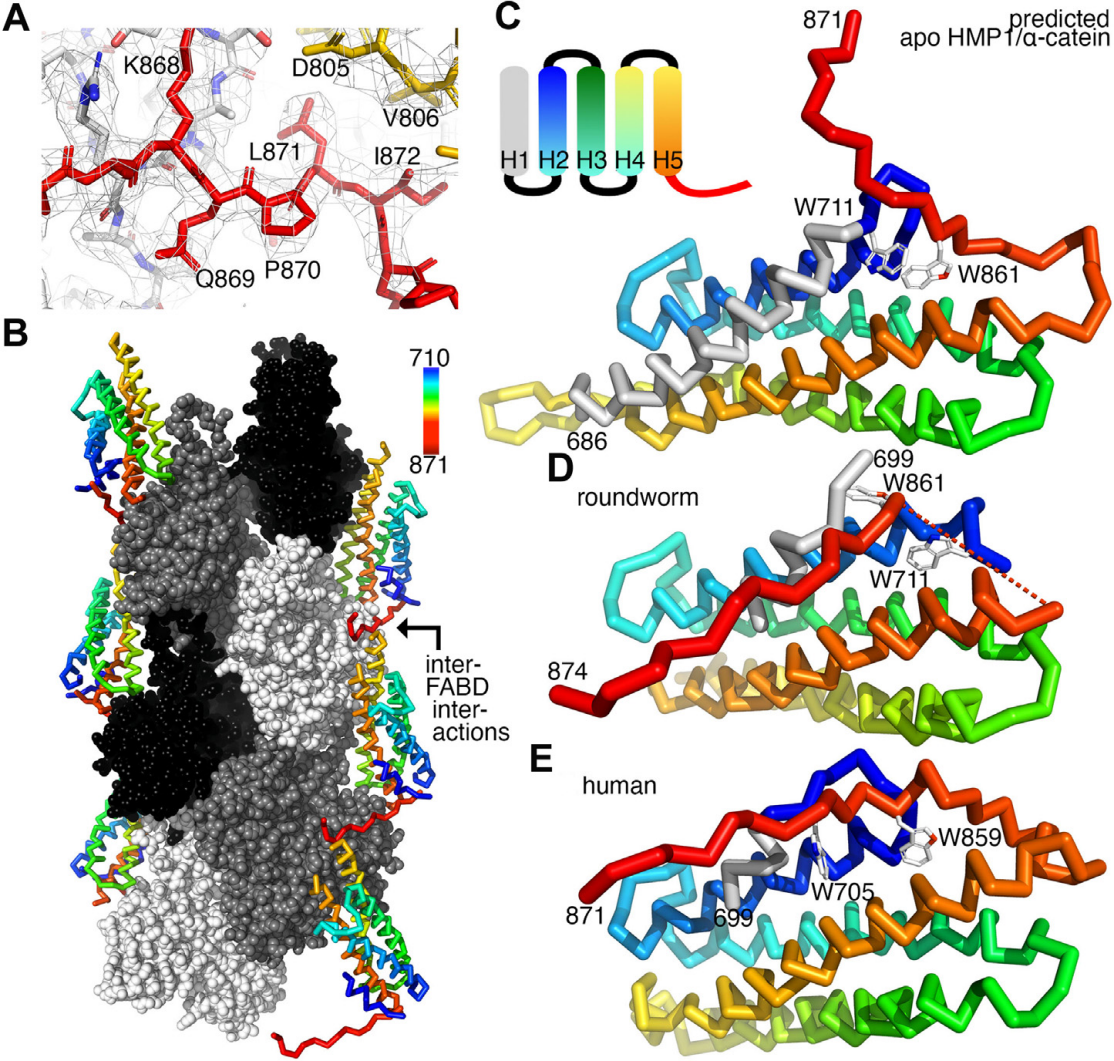
**Cryogenic electron microscopy structure of HMP1/α-catenin bound to F-actin**. *A*, Coulomb potential map showing the intermolecular interaction of one HMP1/α-catenin (*red*) with another HMP1/α-catenin (*yellow*). Actin is shown in *white* with oxygen atoms in *red* and nitrogen atoms in *blue*. *B*, Cα trace of HMP1/α-catenin, colored spectrally from shorter to longer wavelengths for residues 710 to 871 as indicated. Actin subunits are shown as *black*, *gray*, or *white* spheres to distinguish the individual actin polypeptide chains. The F-actin barbed (+) end is at the bottom, and the F-actin pointed end is at the *top* (−). The intermolecular HMP1/α-catenin-HMP/α-catenin interaction is indicated by the *arrow*. *C*, schematic of the five-helix FABD HMP1/α-catenin model as predicted by AlphaFold (32), color-coded as in panel (*B*). Tryptophan residues 711 and 861 are shown. *D*, HMP1/α-catenin cryoEM structure in its F-actin bound state, color-coded as in panel (*B*) and oriented as in panel (*C*). Residues 718 to 784 can be superimposed with root-mean-square deviation of 0.674 Å for 417 atoms. The broken line connects residues 849 with 861 that are disordered in our structure. Note (i) Trp-861 that is swung out compared to the unbound structure in panel (*C*), (ii) the extension of α-helix H2 (*blue*) that occupies the space of the loop (residues 849 to 861) in the unbound structure (shown in panel *C*), and (iii) Trp-711 that fills the pocket that is occupied in the unbound structure (shown in panel C) by Trp-861. *E*, in the human α-catenin structure bound to F-actin (PDB entry 6upv) (18), the Trp-705 (shown in a *stick*) that corresponds to roundworm Trp-711 is further away (shown in panel *D*). FABD, F-actin binding domain.

On the other hand, the isolated HMP1/α-catenin FABD bundles F-actin (Fig. 2) which hampers single particle helical reconstruction. We eventually overcame the challenges that bundling posed for cryoEM structure determination by first applying F-actin on our cryoEM grid, followed by adding HMP1/α-catenin versus using both proteins simultaneously on our cryoEM grid. This process eventually succeeded in our 3.4 Å cryoEM structure determination of HMP1/α-catenin bound to F-actin (Fig. 3 and Table 2). While HMP1/α-catenin was disordered beyond residue 874 or before residue 689 and in three loop regions (residues 699–710, 811–816, and 849–861) the density is otherwise unambiguous (Fig. 4*A*).

**Table 2:** Cryogenic electron microscopy data processing and refinement statistics

	(A) HMP1/α-catenin	(B) F-actin bound HMP1/α-catenin
Microscope	JEOL cryoARM300	JEOL cryoARM300
kV	300	300
Detector	GATAN K3	GATAN K3
Data collection		
Magnification	60,000	60,000
Total dose (e^-^*per* Å^2^)	60	60
Fractions	50	50
Pixel size (Å *per* pixel)	0.72	0.72
Defocus range	−0.6 to −2.6 μm	−0.6 to −2.6 μm
Data processing		
Number of micrographs	1,294	3576
Number of particles	149,802	1,990,015
Resolution	4.8 Å	3.36 Å
Helical rise		27.72 Å
Helical twist		−166.9°
Model refinement statistics		
Polypeptide chains		19
Number of non-H atoms		24,852
Protein residues		3174
Magnesium ions		6
Bonds (root-mean-square deviation)		
Length (no outliers)		0.010 Å
Angles (no outliers)		0.900°
Molprobity score		1.78
Clash score		6.35
Ramachandran plot		
Outliers		0
Allowed		0.0291
Favored		0.9709
Rotamer outliers		0.0223
Cβ outliers		0
CaBLAM outliers		0.0039
EMDB identifier	EMD-26748	EMD-26805
PDB identifier		7uuw

Data and model refinement statistics for (A) unbound HMP1/α-catenin and (B) F-actin bound to the HMP1/α-catenin.

As seen for the binding of mammalian α-catenin, the carboxy terminus of HMP1/α-catenin extends to engage in hydrophobic interactions with HMP1/α-catenin of a neighboring HMP1/α-catenin (Fig. 4*B*). For example, Val-809 of one HMP1/α-catenin interacts with Ile-872 of a separate HMP1/α-catenin polypeptide chain.

There is no structural data available for the FABD of HMP1/α-catenin, and our F-actin bound FABD is the first report of this functionally important domain. Therefore, to understand the potential allostery, we are using the unbound HMP1/α-catenin model as predicted by AlphaFold (32) (Fig. 4*C*). As seen with our human α-catenin crystal structure (27), the FABD of HMP1/α-catenin is a five-helix bundle domain. The five α-helices of this unbound HMP1/α-catenin FABD structure are as seen in our unbound human α-catenin crystal structure (27). Residues 669 to 861 of each human α-catenin protomer of the human α-catenin dimer can be superimposed with root-mean-square deviation of 0.704 Å or 0.603 Å for 869 (chain A) or 920 atoms (chain B), respectively, onto the predicted HMP1/α-catenin model. Therefore, the predicted HMP1/α-catenin FABD structure seems to be a valuable model for the structure of the unbound HMP1/α-catenin FABD.

Major novel F-actin-induced structural changes in the HMP1/α-catenin FABD seem to occur at the amino terminus of the HMP1/α-catenin FABD structure (Fig. 4, *C* and *D*). Specifically, Trp-711 predicted to be at the end of the first α-helix in the unbound HMP1/α-catenin FABD structure (Fig. 4*C*), that is also seen in our unbound human α-catenin structure (27), is instead at the carboxy terminus of the second α-helix in our F-actin bound HMP1/α-catenin structure (Fig. 4*D*). When bound to F-actin, Trp-711 fills the pocket that Trp-861 occupies in the unbound model. HMP1/α-catenin residue Trp-861 corresponds to human α-catenin residue Trp-859 (Fig. S1*B*). In the F-actin bound human α-catenin structure (Fig. 4*E*), Trp-859 fills the pocket similarly as for the equivalent HMP1/α-catenin Trp-861 (Fig. 4*C*). The extension of the second α-helix, by borrowing residues from the loop region, no longer leaves room for the loop region of the last α-helix that therefore becomes disordered in the actin-bound HMP1/α-catenin structure (Fig. S2). Despite these distinct allosteric changes in the amino-terminal roundworm residue 716 (corresponding to human residue 710) that are not in contact with actin, residues 710 to 842 of human actin-bound α-catenin can be superimposed onto the equivalent 694 atoms in our roundworm structure with root-mean-square deviation of 0.699, in agreement with the high degree of sequence identity (39%) and homology (60%) (Fig. S1). Collectively, these helical rearrangements seem unique to the nematode HMP1/α-catenin.

### HMP1/α-catenin is a flexible monomer

**Figure 5:**
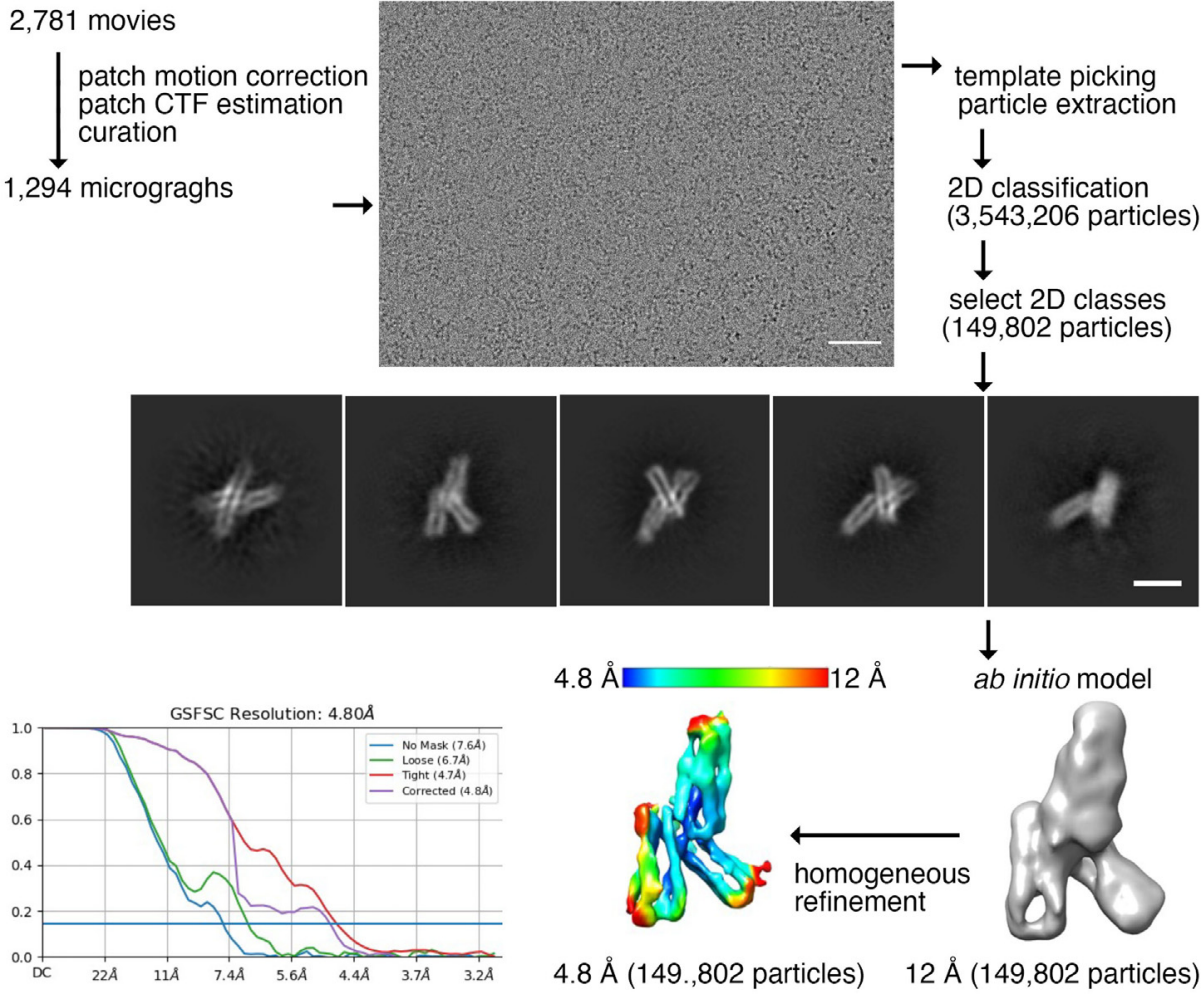
**Cryogenic electron microscopy data processing workflow for HMP1/α-catenin.** A representative motion corrected, contrast transfer function estimated, micrograph is shown (*top*) with select 2D classes (*middle*). The scale bar on the micrograph corresponds to 50 nm. The low resolution (12 Å) *ab initio* model (*bottom right*) along with the final refined 3D reconstruction (*bottom middle*) are depicted. The final resolution of 4.8 Å obtained based on the Fourier shell correlation cut-off value of 0.143 is also provided. The scale bars on the micrographs (*top*) correspond to 50 nm. The scale bar on the 2D class average (*middle*) corresponds to 5 nm. CTF, contrast transfer function.

**Figure 6:**
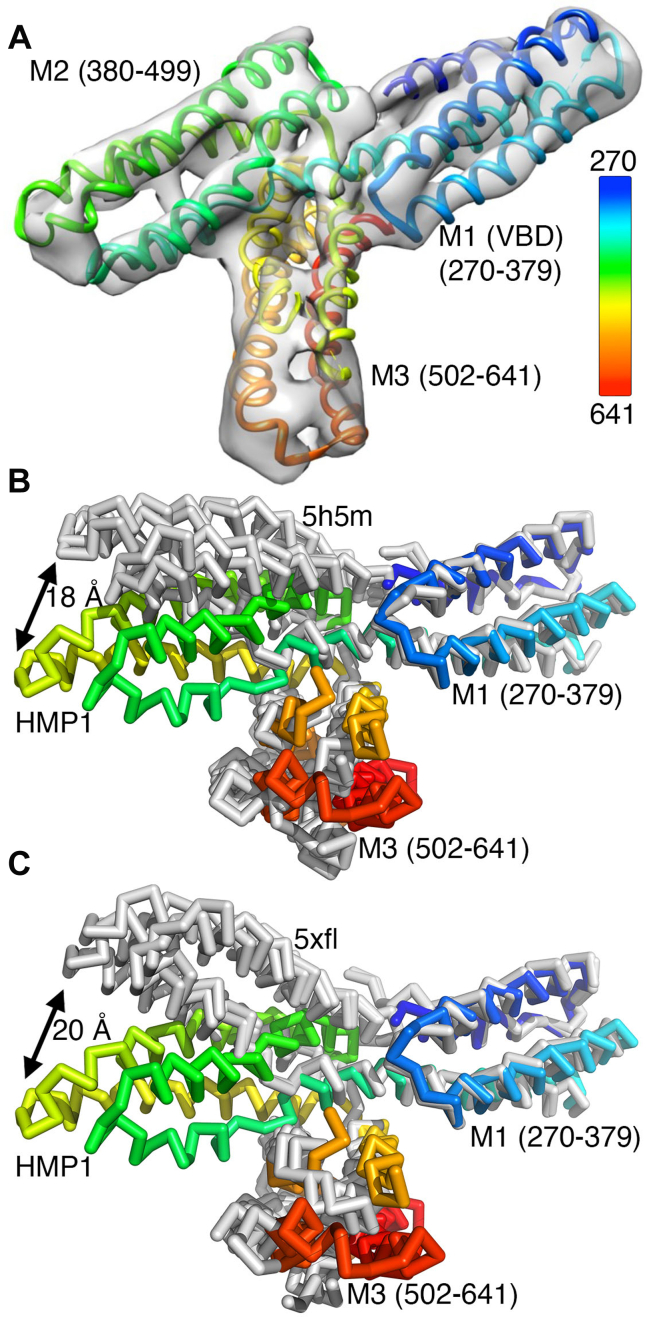
**HMP1/α-catenin has unbound amino- and carboxy-terminal domains and unique middle subdomain orientations.***A*, the 3D reconstruction as obtained from the 4.8 Å resolution cryoEM map is depicted with an overlay of the ribbon representation of our HMP1/α-catenin cryoEM structure showing the middle domain (residues 270–641) colored spectrally from shorter to longer wavelengths for residues 270 to 641. VBD, vinculin binding domain; M1, M2, M3, middle subdomains 1 through 3. *B*, Cα-trace of the truncated HMP1/α-catenin crystal structure (Δ1-269 and Δ647–927, PDB entry 5h5m) (30) superimposed onto our HMP1/α-catenin M1 subdomain cryoEM structure. The two molecules in the asymmetric unit are shown in *gray*. The M1 domain of our full-length HMP1/α-catenin structure, colored spectrally as in panel (*A*), is superimposed and highlights the relative movement (of about 18 Å) of the M2 subdomain (*arrow*). The relative movement of over 8 Å of subdomain M3 is also apparent. *C*, Cα-trace of the truncated murine αN-catenin structure (Δ1-259 and Δ631–953, PDB entry 5xfl) (35) superimposed onto our HMP1/α-catenin M1 subdomain cryoEM structure. The two αN-catenin molecules in the asymmetric unit are shown in *gray*. The M1 domain of our full-length HMP1/α-catenin structure, colored spectrally as in panel (*A*), is superimposed and highlights the relative movement (of about 20 Å) of the M2 subdomain (*arrow*). The relative movement of over 6 Å of subdomain M3 is also apparent.

While vertebrate α-catenins are monomeric or dimeric depending on their location and function, HMP1/α-catenin does not dimerize (Fig.1*B* and Table 1). To determine the effects of the quaternary structure on α-catenin functions, we determined the cryoEM structure of the constitutive monomeric HMP1/α-catenin (Figs. 5, 6 and Table 2). Disappointing at first glance, our full-length HMP1/α-catenin sample only showed cryoEM density for the middle domain, which explains the obtained 4.8 Å resolution despite countless efforts to improve the resolution and conformational heterogeneity such as treatment with glutaraldehyde. Nevertheless, this structure determination is a substantial achievement especially since attempts to determine the full-length monomeric murine αN-catenin structure to just 6.5 Å also had its amino-terminal and carboxy-terminal domains disordered (33). Further, the wealth of available crystal and predicted structures (32) allows significant insights even at this modest resolution.

Specifically, in our full-length HMP1/α-catenin sample, the remaining domains (the amino-terminal domain, residues 1–269, and the carboxy-terminal FABD, residues 642–927) were not detected in 2D classification. This suggests that the middle domain of HMP1/α-catenin is the only part rigid enough to align during 2D classification, while the rest of HMP1/α-catenin is flexible (Fig. 6*A*). This disorder is consistent with SEC-small-angle X-ray scattering (SAXS) data that showed that the FABD connects *via* a flexible linker (residues 630–675) to the middle domain (34).

In comparison to our cryoEM HMP1/α-catenin structure, the truncated HMP1/α-catenin crystal structure (Δ1-269 and Δ647–927, PDB entry 5h5m) (30) has two of the three four-helix bundles that make up the middle domain oriented differently probably due to crystal contacts (Fig. 6*B*). Similarly, the truncated murine αN-catenin structure (Δ1-259 and Δ631–953, PDB entry 5xfl) (35) also has two of the three 4-helix bundles that make up the middle domain oriented differently probably due to crystal contacts (Fig. 6*C*). While the truncated HMP1/α-catenin crystal structure has one polypeptide chain in the asymmetric unit (space group *P* 2_1_2_1_2_1_), the truncated αN-catenin structure has two polypeptide chains in the monoclinic (*P* 2_1_) crystal. Thus, despite the different crystal contacts in the nematode *versus* murine middle domain structures, these can be superimposed with 1.8 Å root-mean-square deviation for 1,647 atoms. Given that the two crystal lattices resulted in similar interdomain interactions, it seems that in full-length HMP1/α-catenin, the interdomain interaction differs by ∼20 Å movements, as seen in our cryoEM structure (Fig. 6, *B* and *C*).

### Nematode and human α-catenin binding alters the F-actin structure

The smallest and most flexible of the four actin domains harbors the D-loop (for DNase I binding loop; residues 40–51) that mediates longitudinal actin-actin interactions (36, 37). Adenosine triphosphate hydrolysis causes conformational changes of the D-loop conformation. Binding of HMP1/α-catenin results in allosteric changes in the D-loop of F-actin that were induced by the binding of human (PDB entry 6upv) or murine (PDB entry 6wvt) α-catenin to F-actin (17, 18). Specifically, 2632 (or 2555) HMP1/α-catenin bound actin atoms can be superimposed with root-mean-square deviation of 0.583 Å or 0.789 Å for F-actin bound to human (PDB entry 6upv) or murine (PDB entry 6wvt) α-catenin, respectively (Fig. S3).

## Discussion

Molecular insights into α-catenin are crucial to understanding α-catenin regulation of cell–cell contacts. While a wealth of mammalian α-catenin structural data is available, structural insights into HMP1/α-catenin are limited. Thus, our HMP1/α-catenin studies provide significant new structural data and will additionally aid our understanding of the evolution of multicellularity in metazoans.

Despite the high sequence identity amongst α-catenins from different species, only some of these proteins dimerize. Our full-length HMP1/α-catenin cryoEM structure shows that HMP1/α-catenin is a highly flexible monomeric protein. Several phosphorylation sites have been identified in HMP1/α-catenin (31). In contrast to the phosphomimetic S509E mutant, the nonphosphorylatable S509A mutant displayed morphological defects (38). In our HMP1/α-catenin cryoEM structure, Ser-509 is part of the third four-helix bundle subdomain M3 of the HMP1/α-catenin middle domain and engages in close interdomain contacts with the first four-helix bundle subdomain M1 of the HMP1/α-catenin middle domain. Our structure suggests that a phosphorylated Ser-509 would open up the HMP1/α-catenin middle domain and sever the M1-M3 interdomain interactions. Since M1 is the vinculin binding domain (39, 40), this might be consequential for the binding of HMP1/α-catenin to DEB1/vinculin. Notably, Ser-509 is conserved in vertebrates.

In contrast, the phosphomimetic and nonphosphorylatable mutant of Ser-649 localized to cell–cell junctions as seen for wild type HMP1/α-catenin middle domain (38). This is consistent with structural data since Ser-649 is part of the linker region between the HMP1/α-catenin middle domain and the HMP1/α-catenin FABD.

The lack of interdomain interactions in our full-length HMP1/α-catenin is relevant to its functions. First, the amino-terminal HMP1/α-catenin domain that binds HMP2/β-catenin at cell–cell junctions is disordered in our structure and therefore displays considerable conformational freedom. This allows HMP1/α-catenin to readily bind to partners like HMP2/β-catenin without prior activation. Similarly, we find that the carboxy-terminal FABD of HMP1/α-catenin is not interacting with the other HMP1/α-catenin domains. In contrast to mammalian α-catenin, HMP1/α-catenin is therefore constitutively active in linking HMR1/cadherin to the actin cytoskeleton. Indeed, there are also differences in the affinity of the various α-catenins with filamentous actin (13, 41).

SEC-SAXS data showed that monomeric human αE-catenin is a compact globular structure with a maximum dimension of about 147 Å (42) that is indicative of a compact multidomain protein (43). This dimension determined by SEC-SAXS is comparable to the size of one protomer in our human α-catenin dimer in our crystal structure (27). SEC-SAXS data from another group also showed at least an interdomain interaction between the amino-terminal and the middle domains of human α-catenin (34). Therefore, roundworm HMP1/α-catenin seems more flexible compared to the mammalian α-catenins.

Human α-catenin was shown to bind F-actin cooperatively (22, 44, 45). As seen in the cryoEM structures of human α-catenin bound to F-actin, the carboxy terminus of HMP1/α-catenin interacts with another HMP1/α-catenin polypeptide chain. This interaction likely plays a role in cooperative binding to F-actin and seems independent of the amino-terminal domain. It is worth noting, that cooperative binding of α-catenin is decreased in the full-length protein compared to just the FABD (44).

We found that full-length HMP1/α-catenin co-sedimented with filamentous actin. A S823F mutant has previously been shown to reduce the binding of HMP1/α-catenin with F-actin (38). This is consistent with our actin-bound HMP1/α-catenin structure where the hydroxyl of the HMP1/α-catenin residue Ser-823 is within 4 Å to the amide of the F-actin residue Arg-147. Inspection of our actin-bound HMP1/α-catenin cryoEM structure suggests that the larger hydrophobic phenylalanine side chain would be difficult to accommodate.

Furthermore, the isolated HMP1/α-catenin FABD resulted in heavy F-actin bundling, as seen by our cryoEM analyses. Such bundling was not observed by full-length HMP1/α-catenin. Our findings are consistent with an earlier report that showed that HMP1/α-catenin co-sedimented with F-actin significantly less compared to the isolated HMP1/α-catenin FABD (38). Our cryoEM data show how the HMP1/α-catenin FABD bridges two actin filaments.

The carboxy terminus is disordered in our F-actin-bound FABD HMP1/α-catenin cryoEM structure and in our dimeric full-length human crystal structure. Given that carboxy-terminal truncations prevent bundling, it seems that the last ∼20 residues are responsible for bundling. The AlphaFold (32) model of HMP1/α-catenin predicts residues 891 to 927 to be helical. This opens the possibility of forming an anti-parallel coiled coil with a neighboring FABD as the molecular mechanism of bundling.

It is tempting to speculate that in the full-length monomeric structure, this carboxy-terminal region might be buried in intermolecular interactions and thus prevent bundling.

Although our actin-bound sample for cryoEM structure determination comprises HMP1/α-catenin residues 677 to 927, the HMP1/α-catenin amino terminus (residues 677–710) was disordered in our cryoEM map. This is consistent with the finding that HMP1/α-catenin Δ677-703 localized to cell–cell junctions as seen for wild type HMP1/α-catenin (38). However, it is unclear what the consequences are to the structural integrity of HMP1/α-catenin upon deletion of residues 677 to 703.

While mammalian α-catenins are autoinhibited and regulated by mechanical force, these effects in *C. elegans* are just beginning to be uncovered (20). The helical remodeling of the first FABD HMP1/α-catenin α-helix that was revealed by our cryoEM structures and seems unique to HMP1/α-catenin, might be a key element in the regulation of roundworm *versus* mammalian α-catenins in particular to the regulation by mechanical force.

## Experimental procedures

### Cloning and protein production

Full-length cDNA of *Danio rerio* was obtained from GE Healthcare Dharmacon Inc., human α-catenin from Open Biosystems, and *C. elegans* HMP1/α-catenin were kindly provided by Dr Yuji Kohara, Center for Genetic Resource Information, National Institute of Genetics, Research Organization of Information and Systems, Mishima, Japan. These cDNAs were used as templates to subclone into pGEX-6P-1 using the restriction-free cloning method (46). The glutathione S-transferase (GST)-tagged *D. rerio* α-catenin and HMP1/α-catenin constructs have a PreScission protease cleavage site immediately after the GST tag. The set of primers used for cloning are as follows:

pGEX-6P-1 vector:

Forward: TGAGGTCGACTCGAGCGGCCGC

Reverse: GGATCCCAGGGGCCCCTGGAACAGAACTTCCAG

HMP1/α-catenin:

Forward: GGGCCCCTGGGATCCATGCCTGCGAATGGCAATTCTCATGCG

Reverse: GCCGCTCGAGTCGACCTCATAAACGACCGTTTATTCTTTGTTGATGCC

*D. rerio* α-catenin:

Forward: GGGCCCCTGGGATCCATGACGAGCATTAACACTGCTAACATCAACTTC

Reverse: GCCGCTCGAGTCGACCTCAAATACTATCCATAGCTTTGAACTCGCTCAGGG

The human α-catenin FABD (residues 660–906, 660–885, or 660–865) were cloned into the pET28 vector to have an amino-terminal octa-Histidine tag followed by a PreScission protease site immediately before the inserts using the following primers:

pET28 vector:

Forward: TGAGAATTCGCGGCCGCACTCGAGCACCAC

Reverse: CATATGGGGCCCCTGGAACAGAACTTCCAGATG

Human α-catenin FABD (upper case is used for the vector and lower case for the insert):

660 Forward: CTGGAAGTTCTGTTCCAGGGGCCCcatgacgatcagctgatagctggcc

906 Reverse: CGGCCGCGAATTCTCAgatgctgtccatagctttgaactcgctg

885 Reverse: CGGCCGCGAATTCTCAtgcccgtttaatcttggtctgtgtctcatc

865 Reverse: CGGCCGCGAATTCTCActctggtgccttcatcttccatg

The HMP1/α-catenin FABD (residues 677–927) and full-length human α-catenin (residues 1–906) were cloned into pGEX-6P-1 with an amino-terminal GST tag and a PreScission protease cleavage site by DNA Custom Cloning. The expression construct of *D. discoideum* α-catenin was procured as an amino-terminally tagged GST fusion with a tobacco etch virus cleavage site (Dicty stock center).

Constructs were transformed into BL21(DE3) (Novagen) and expressed in 2 L of Luria-Bertani media at 37 °C until the absorbance at 600 nm reached 0.6 to 0.8 and induced with 1 mM isopropyl β-D-1-thiogalactopyranosides for 20 h at 25 °C. Cells were pelleted by centrifugation at 5000*g* for 15 min and stored at −80 °C.

### Protein purification

To generate purified full-length HMP1/α-catenin, frozen *Escherichia coli* BL21(DE3) pellets containing amino-terminally GST-tagged HMP1/α-catenin were thawed and resuspended in lysis buffer containing 400 mM NaCl, 20 mM tris(hydroxymethyl)aminomethane (pH 8), 0.5 mM ethylenediaminetetraacetic acid (EDTA), 2 mM β-mercaptoethanol, and 1 mM phenylmethylsulfonyl fluoride, and lysed by sonication (75% amplitude with 3 min processing time and 5 s on followed by 10 s off). The lysate was clarified by centrifugation (35,000*g* for 30 min at 4 °C) and then loaded onto a GST prep 16/10 FF chromatography column that was equilibrated with 150 mM NaCl, 20 mM tris(hydroxymethyl)aminomethane (pH 8), 0.5 mM EDTA, and 2 mM β-mercaptoethanol. Elution was completed with 150 mM NaCl, 20 mM tris(hydroxymethyl)aminomethane (pH 8), 0.5 mM EDTA, 2 mM β-mercaptoethanol, and 10 mM reduced glutathione. Peak elution fractions were pooled and dialyzed overnight in 2 L of buffer containing 150 mM NaCl, 20 mM tris(hydroxymethyl)aminomethane (pH 8), 0.5 mM EDTA, and 2 mM β-mercaptoethanol, after the addition of 200 μg PreScission protease. The sample was then reloaded onto a GST prep 16/10 FF column. The flowthrough containing cleaved HMP1/α-catenin was collected, concentrated to 2 mg/ml, and aliquoted into 1 ml aliquots after adding glycerol (10% final concentration), and stored at −80 °C.

Prior to cryoEM grid preparation, an aliquot was thawed and passed through a Superdex S200 10/300 Gl column equilibrated in 150 mM NaCl, 20 mM tris(hydroxymethyl)aminomethane (pH 7.5), and 1 mM dithiothreitol. The purification of the FABD of HMP1/α-catenin was performed as described for full-length HMP1/α-catenin.

Full-length human, *D. rerio*, and *D. discoideum* α-catenins were purified similar to HMP1/α-catenin using 1 mM dithiothreitol instead of β-mercaptoethanol. Cleavage of the GST tag for *D. discoideum* α-catenin was carried out with tobacco etch virus protease.

For full-length human α-catenin, the monomer and dimer peaks were pooled separately, and a second SEC run was performed of each pool on a Superdex S200 10/300 Gl column pre-equilibrated in 20 mM tris(hydroxymethyl)aminomethane (pH 8), 150 mM NaCl, and 0.2 mM tris(2-carboxyethyl)phosphine.

For purification of the human α-catenin FABD proteins (residues 660–906, 660–885, or 660–865), frozen *E. coli* BL21(DE3) pellets containing amino-terminally octa-Histidine-tagged α-catenin FABDs were thawed, resuspended in 500 mM NaCl, 20 mM tris(hydroxymethyl)aminomethane (pH 8), and 1 mM β-mercaptoethanol, and lysed by sonication for 3 min (5 s on and 10 s off cycles). The lysate was clarified by centrifugation at 100,000*g* for 45 min and filtered. The supernatant was loaded onto two HisTrap HP 5 ml columns in tandem equilibrated in a buffer containing 150 mM NaCl and 20 mM tris(hydroxymethyl)aminomethane (pH 8) on an AKTA FPLC at a flow rate of 1 ml/min. The column was then washed thoroughly with 30 column volumes of buffer containing 150 mM NaCl, 20 mM tris(hydroxymethyl)aminomethane (pH 8), and 50 mM imidazole. Bound protein was then eluted using a gradient to 500 mM imidazole. The eluted protein fractions were pooled and subjected to overnight PreScissoin protease treatment (200 μg) with dialysis against a buffer containing 150 mM NaCl, 20 mM tris(hydroxymethyl)aminomethane (pH 8), 1 mM ethylenediaminetetraacetic acid, and 1 mM dithiothreitol. The PreScissoin protease treated α-catenin FABD proteins were concentrated and loaded onto a Superdex 75 26/60 column equilibrated in 150 mM NaCl, 20 mM tris(hydroxymethyl)aminomethane (pH 8), and 1 mM dithiothreitol. The peak fractions were concentrated and stored at −80 °C.

### Size exclusion chromatography and multiangle light scattering

We used SEC and multiangle light scattering analyses to determine the absolute masses with an Agilent 1260 Infinity HPLC with a variable wavelength detector for UV absorption monitoring which is coupled in line with Dawn-Heleos II multiangle light-scattering detector (Wyatt Technology) and OptiLab T-rex differential refractive index detector (Wyatt Technology). We loaded our samples on an analytical Superdex S200 10/300 Gl column that was pre-equilibrated with 20 mM tris(hydroxymethyl)aminomethane (pH 8), 150 mM NaCl, and 0.2 mM tris(2-carboxyethyl)phosphine, at a flow rate of 0.5 ml/min. We used bovine serum albumin (Pierce) as a standard and performed data acquisition and analyses using ASTRA software version 6.1.

### Actin polymerization

We dialyzed rabbit skeletal muscle G-actin (50 μl at 1 mg/ml) into 500 ml of G-actin buffer (2 mM tris(hydroxymethyl)aminomethane [pH 8], 0.2 mM adenosine triphosphate, 0.2 mM CaCl_2_, 0.5 mM β-mercaptoethanol) at 4 °C. We polymerized G-actin (at 0.6 mg/ml) by adding the polymerization buffer (50 mM KCl, 2 mM MgCl_2_, 10 mM imidazole, 2 mM adenosine triphosphate, and 5 mM ethylene glycol tetraacetic acid) to fully polymerize overnight. F-actin was stored at 4 °C for up to 2 weeks.

### Actin co-sedimentation

For F-actin co-sedimentation assays, rabbit muscle actin (Cytoskeleton Inc) was freshly polymerized either in Tris buffer (20 mM tris(hydroxymethyl)aminomethane [pH 8.0], 0.2 mM CaCl_2_, 10 mM MgCl_2_, 100 mM KCl, and 10 mM adenosine triphosphate) for binding assays or in KMEI buffer (50 mM KCl, 2 mM MgCl_2_, 10 mM imidazole (pH 7), 2 mM adenosine triphosphate, and 0.5 mM ethylene glycol tetraacetic acid) and used for bundling assays. For binding assays, F-actin (15 μM) was incubated with 8 μM full-length proteins of HMP1/α-catenin, zebrafish (*D. reiro*) α-catenin, or slime mold (*D. discoideum*) α-catenin. For F-actin bundling assays, F-actin (4 μM) was incubated with 3 μM of full-length HMP1/α-catenin or 15 μM of HMP1/α-catenin FABD proteins. After incubation at room temperature for 15 min, the samples were spun at appropriate speeds (100,000*g* for binding or 15,000*g* for bundling) for 15 min. The supernatant and the pellet were analyzed on sodium dodecyl-sulfate polyacrylamide gel electrophoresis and the bands visualized by Coomassie R-250 staining. As controls, F-actin bundling with full-length α-catenin (residues 1–906) monomer and dimer along with α-catenin FABDs (residues 660–906, 660–885, and 660–865) were also analyzed.

### CryoEM grid preparation

For the HMP1/α-catenin FABD in complex with F-actin cryoEM structure determination, C-Flat 1.2/1.3400 mesh grids were glow discharged for 60 s at 15 mA using a PELCO easiGlow (Ted Pella Inc) and mounted on Leica GP2 cryogenic plunger (65% humidity and 21 °C). First, 3.5 μl of F-actin (0.7 μM) was placed on the carbon film and incubated for 60 s and subsequently successive addition and removal of 3.5 μl of 0.6 mg/ml (21 μM) HMP1/α-catenin. The addition and removal steps were repeated twice before blotting from the backside of the grid for a total of 12 s. The grids were immediately plunge frozen in liquid ethane that is maintained at -183 K and cooled by liquid nitrogen. Lower concentrations of the HMP1/α-catenin FABD were unsuitable as they resulted in partial decoration. The grids were transferred into a Japan Electron Optics Laboratory (JEOL) cryoARM300 for screening and data collection.

For the full-length HMP1/α-catenin cryoEM structure determination, an Au-Flat 1.2/1.3300 mesh grid (Protochips) was glow discharged at 15 mA for 240 s using a PELCO easiGlow (Ted Pella Inc) and taken into Leica GP2 cryogenic plunger set to 95% humidity and 4 °C. About 4 μl of 0.3 mg/ml of full-length HMP1/α-catenin was applied on the carbon film side, blotted on the carbon side for 7 s, and plunged immediately for vitrification in liquid ethane and transferred into a JEOL cryoARM300.

### CryoEM data collection

All cryoEM data were recorded on a JEOL cryoARM300 operated at 300 kV. The illumination was set to a spot size of three and an angle of four at a magnification of 60,000. The condenser aperture was set to 50 μm with an Omega in-column energy filter, a slit width of 20 eV, and a zero-loss peak that aligned every 6 h. Electron micrographs were collected using serialEM (47) on a K3 detector (Gatan) set in correlated double sampling mode and a calibrated object pixel size of 0.72 Å. The corresponding flux of the detector was seven electrons/pixel/second.

### CryoEM image processing

Raw movies were imported into the cryoSPARC (48) interface. Motion correction was accomplished by patch motion correction (49) and contrast transfer function (CTF) estimation with a CTF patch estimation. Curation of micrographs triaged (i) all micrographs that had a CTF fit greater than 3.5 Å for the F-actin contained samples or 5 Å for full-length HMP1/α-catenin sample and a total full-frame motion distance over 150 pixels, (ii) astigmatic micrographs, and (iii) micrographs with thick or crystalline ice. For HMP1/α-catenin bound to F-actin, the Filament Tracer function picked particles with a template free tracing with 50 Å and 500 Å for minimum and maximum filament diameter, respectively, as a criterion to select as many particles as possible and remove unusable particles during the initial 2D classification.

For the FABD of HMP1/α-catenin bound to F-actin, 20,737 micrographs were collected to compensate for the high degrees of bundling observed in many micrographs and to improve the final resolution. After micrograph curation, 3576 images were selected, while the rest were removed. Since the 2D classes exhibited a high degree of decoration, a larger box size was not needed to separate unbound from bound particles. Instead, an extraction box size of 1080 pixels (399 Å) was used, and 2D classification was only used to separate suitable particles from unsuitable particles. After helical refinement, CTF refinement, and nonuniform refinement, a reconstruction of a 3D map at 3.36 Å resolution from 1,990,015 particles was achieved (EMDB entry EMD-26805), with a helical twist of −166.9°, and a helical rise of 27.72 Å.

For full-length HMP1/α-catenin, a total of 2781 movies were collected, and after curation, 1294 micrographs were selected for further processing. Select 2D classes obtained from the initial round of blob picking and extraction were used to carry out the second round of template-based particle picking. The resultant 2D classes were used to select suitable particles for initial *ab initio* volume calculation. The final homogenous refinement yielded a 4.8 Å resolution reconstruction from 149,802 particles (EMDB entry EMD-26748).

### Structure determination

To determine the cryoEM structure of the HMP1/α-catenin FABD bound to F-actin, the cryoEM structure of our human α-catenin (residues 22–906) bound to F-actin was first fitted into the map using Chimera. A HMP1/α-catenin homology model was generated with SWISS-MODEL (50) and then docked into the cryoEM map density. The structure was then modified to fit the map to the best of our ability using COOT (51) and further refined through PHENIX (52, 53) using simulated annealing and then rigid body real-space refinement, after which outliers were inspected and corrected. The final structure was deposited with the PDB (entry 7uuw).

To determine the cryoEM structure of full-length HMP1/α-catenin, the crystal structure of the middle domain of HMP1/α-catenin (PDB entry 5h5m) was initially docked into our cryoEM map. The structure was better fitted after chain refinement using COOT (51).

## Data availability

The coordinates for the HMP1/α-catenin FABD bound to F-actin are deposited with the Protein Data Bank, accession code 7uuw. The cryoEM reconstructions for the HMP1/α-catenin FABD bound to F-actin and the unbound HMP/α-catenin were deposited in EMDB with accession codes EMD-26805 and EMD-26748, respectively. All other data are within the manuscript.

## Supporting information

This article contains supporting information (10, 18, 28, 54).

## Conflict of interest

The authors declare no conflict of interest with the contents of this article.
