# Supplementary material for: The nematode α-catenin ortholog, HMP1, has an extended α-helix when bound to actin filaments
Source: J Biol Chem. 2022 Dec 17;299(2):102817. doi: 10.1016/j.jbc.2022.102817 (PMC9860117; doi:10.1016/j.jbc.2022.102817)
Supplement: Supplemental information [file mmc1.pdf]

SUPPLEMENT

The nematode α-catenin ortholog, HMP1, has an extended α-helix when bound to actin filaments

Erumbi S. Rangarajan[1], Emmanuel W. Smith[1] & Tina Izard[1,2]*
* corresponding author; email candice.losey@ufl.edu

From the [1]Cell Adhesion Laboratory, UF Scripps, Jupiter, FL
[2] The Skaggs Graduate School, The Scripps Research Institute

Supplementary Figure S1. Sequence alignment by Protein BLAST (1) of human α-catenin (Hs) and the *Caenorhabditis elegans* ortholog, HMP1/α-catenin (Ce)

The sequence alignment reveals 39% sequence identity and 60% sequence similarity. Similar amino acids are indicated by a plus sign.

(**A**) Sequence alignment of the human (Hs) and roundworm (Ce) amino-terminal and middle domains of α-catenin. Removal of the first 19 human residues (highlighted in yellow) keeps α-catenin dimeric (2). Removal of the first 76 roundworm residues (gray) forces dimerization (3).

```
Hs   1                                           MTAVHAGNINFKWDPKSL 18
Ce   1                                               MPANGNSHAYFNID 14

Hs  19 EIRTLAVERLLEPLVTQVTTLVNTNSKGPSNKKRGRSKKAHVLAASVEQATENFLEKGD- 77
       E+R+  V + +  L+ +VT +  T    P     G       L A+++ A  NFL+ G
Ce  15 EVRSKNVLKQITQLINEVTNITETFPLKPGQTTEG-------LVATLDAAVANFLQTGSF 67

Hs  78 -----KIAKESQFLKEELVAAVEDVRKQGDLMKAAAGEFADDPCSSVKRGNMVRAARALL 132
             IA       + L  A+  V+  G +M     +F  D  S+ KR     + R LL
Ce  68 AISKCPIANSDPRAIDLLHEALGAVQDTGQVMIQTGRDFVRDSTSTNKRAIATNSGRNLL 127

Hs 133 SAVTRLLILADMADVYKLLVQLKVVEDGILKLRNAGN---EQDLGIQ----YKALKPEVD 185
       +AV + LILAD  DV       KV+ D + ++R   +   E D  I+    Y  L  +++
Ce 128 TAVAKFLILADSIDV-------KVIVDKVDEVRETAHQMIEADTKIKVDDLYNLLISQIE 180

Hs 186 KLNIMAAKRQQELKDVGHRDQMAAARGILQKNVPILYTASQACLQHPDVAAYKANRDLIY 245
       +L+I   +R  +L     RD + AAR  L++  P+LYT+++  ++HP+    + NRD
Ce 181 ELDITVRRRAIDLVKPNQRDDLLAARSALRQTAPLLYTSTRTFVRHPEHEEARRNRDYTA 240

Hs 246 KQLQQAVTGISNAAQATASDDASQHQGGGGGELAYALNNFDKQIIVDPLSFSEERFRPSL 305
        ++  A+  + +          +  + G  G+L   ++ F  +I +DP  +     RP L
Ce 241 DEMHSALNALESVLNGQQPK-VTFSEYGRIGDLINEIDTFQNRIEIDPAHYRRGTDRPDL 299

Hs 306 EERLESIISGAALMADSSCTRDDRRERIVAECNAVRQALQDLLSEYMGNAGRKERSDALN 365
       E   E I+SG+A +AD+  TR++R+++IVAECN +RQALQ+LL+EY  + GR++ +D +
Ce 300 EGHCERIVSGSASIADAESTRENRKQKIVAECNNLRQALQELLTEYEKSTGRRDDNDDIP 359

Hs 366 SAIDKMTKKTRDLRRQLRKAVMDHVSDSFLETNVPLLVLIEAAKNGNEKEVKEYAQVFRE 425
         I ++ K+T+DLRR LR+A++DH+SD+FL+T  PL++LIEAAK G+E+  +  +++F+E
Ce 360 LGIAEVHKRTKDLRRHLRRAIVDHISDAFLDTRTPLILLIEAAKEGHEENTRYRSKMFQE 419

Hs 426 HANKLIEVANLACSISNNEEGVKLVRMSASQLEALCPQVINAALALAAKPQSKLAQENMD 485
       HAN+++ VA L+C +S++ E V +++ +A+QLE L PQV  AA+ L  +P SK AQENM+
Ce 420 HANEIVSVARLSCQLSSDVESVSVIQHTAAQLEKLAPQVAQAAILLCHQPTSKTAQENME 479

Hs 486 LFKEQWEKQVRVLTDAVDDITSIDDFLAVSENHILEDVNKCVIALQEK---------DVD 536
        +K  W  +VR+LT A+D+IT++DDFLAVSE HI+ED  + +  +            + +
Ce 480 TYKNAWFDKVRLLTTALDNITTLDDFLAVSEAHIVEDCERGIKGITANASTPDENAANCE 539

Hs 537 GLDRTAGAIRGRAARVIHVVTSEMDNYEPGVYTEKVLEATKLLSNTVMPRFTEQVEA--- 593
        +D  AG+IRGRA RV  VV +EMD  +   YTE V +A ++L    + +F E+  A
Ce 540 TVDCAAGSIRGRALRVCDVVDAEMDFLQNSEYTETVKQAVRILKTQRVDQFAERASALAN 599

Hs 594 --AVEALSSDPAQPMDE-NEFIDASRLVYDGIRDIRKAVLMIRTPEELD-DSDFETEDFD 649
             L+ DP    +E NEFI+A  LV+D ++DIR A+LM R+  ++D D ++E +
Ce 600 RQEAHGLTWDPKTKEEEMNEFINACTLVHDAVKDIRHALLMNRSMNDVDSDVEYEADGVG 659
```

(**B**) Sequence alignment of the human (Hs) and roundworm (Ce) α-catenin FABDs
The structurally distinct tryptophan residues are highlighted in green and cyan.

```
Hs 650 VRSRTSVQTEDDQLIAGQSARAIMAQLPQEQKAKIAEQVASFQEEKSKLDAEVSKWDDSG 709
         +  + +T  +Q    ++ + +M +LP+E+K KI  Q+  F+  +++ + EV+KWD++G
Ce 660 AANADANRTISEQ----ENQQNLMRRLPEEEKKKIQAQIDIFKVTQTRFEREVAKWDETG 715

Hs 710 NDIIVLAKQMCMIMMEMTDFTRGKGPLKNTSDVISAAKKIAEAGSRMDKLGRTIADHCPD 769
       NDII LA  MC IMM MT+FTRG GPLK T DVI AA++I+  GS+++ L R I +   D
Ce 716 NDIISLANNMCKIMMSMTEFTRGCGPLKTTMDVIRAAQEISLNGSKLNALARQIGEESAD 775

Hs 770 SACKQDLLAYLQRIALYCHQLNICSKVKAEVQNLGGELVVSGVDSAMSLIQAAKNLMNAV 829
       S  K+DLLAYL +I LYC QLNICSKVKA+V  +G ELVVS +DSAMSLIQ A+NL+ AV
Ce 776 SQTKKDLLAYLSQITLYCQQLNICSKVKADVTQVGNELVVSALDSAMSLIQTARNLLTAV 835

Hs 830 VQTVKASYVASTKYQKSQGMASLNLPAVSWKMKAPEKKPLVKREKQDETQTKIKRASQKK 889
       VQTVKA+Y+ASTK+++       N   V W+M  P+K+PL++ +K +     I+RAS+++
Ce 836 VQTVKAAYIASTKFRRPNA----NSVRVEWRMAPPKKQPLIRPQKNNAI---IRRASERR 888

Hs 890 HVNPVQALSEFKAMDSI 906
       + P + L+EF
Ce 889 PLQPAKVLAEFTRNEIETGRDSDDEELDRRHQQRINGRL 927
```

(**C**) **Distinct nematode (left) and mammalian (right, PDB entry 6upv)** (4) **α-catenin cryoEM structures in their F-actin-bound state**

The helical bundles that are similar in both structures are shown as Cα-traces in the same orientation. Areas of distinct conformations are shown in stick representation. Key tryptophan residues are labeled in black, and other conformationally distinct residues in gray.

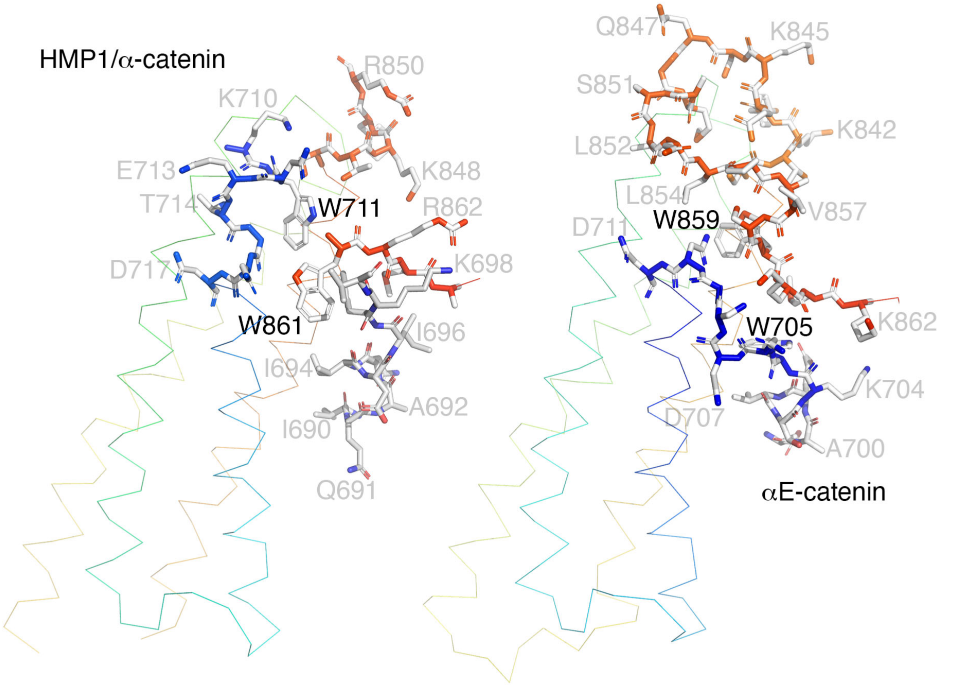

Supplementary Figure S2. Binding of F-actin to the FABD of HMP1/α-catenin extends the second α-helix of this five-helix bundle domain

(**A**) Cα trace of HMP1/α-catenin (colored spectrally from shorter to longer wavelengths for residues 710 to 871) and actin subunits (in gray). The broken line connects residues 849 with 861 which are disordered in our structure. Neighboring actin subunits are labeled as actin and actin'.
(**B**) Cα trace of the human α-catenin structure bound to F-actin (PDB entry 6upv) (4) oriented as our F-actin bound HMP1/α-catenin structure in panel (A). Neighboring actin subunits are labeled as actin and actin'.

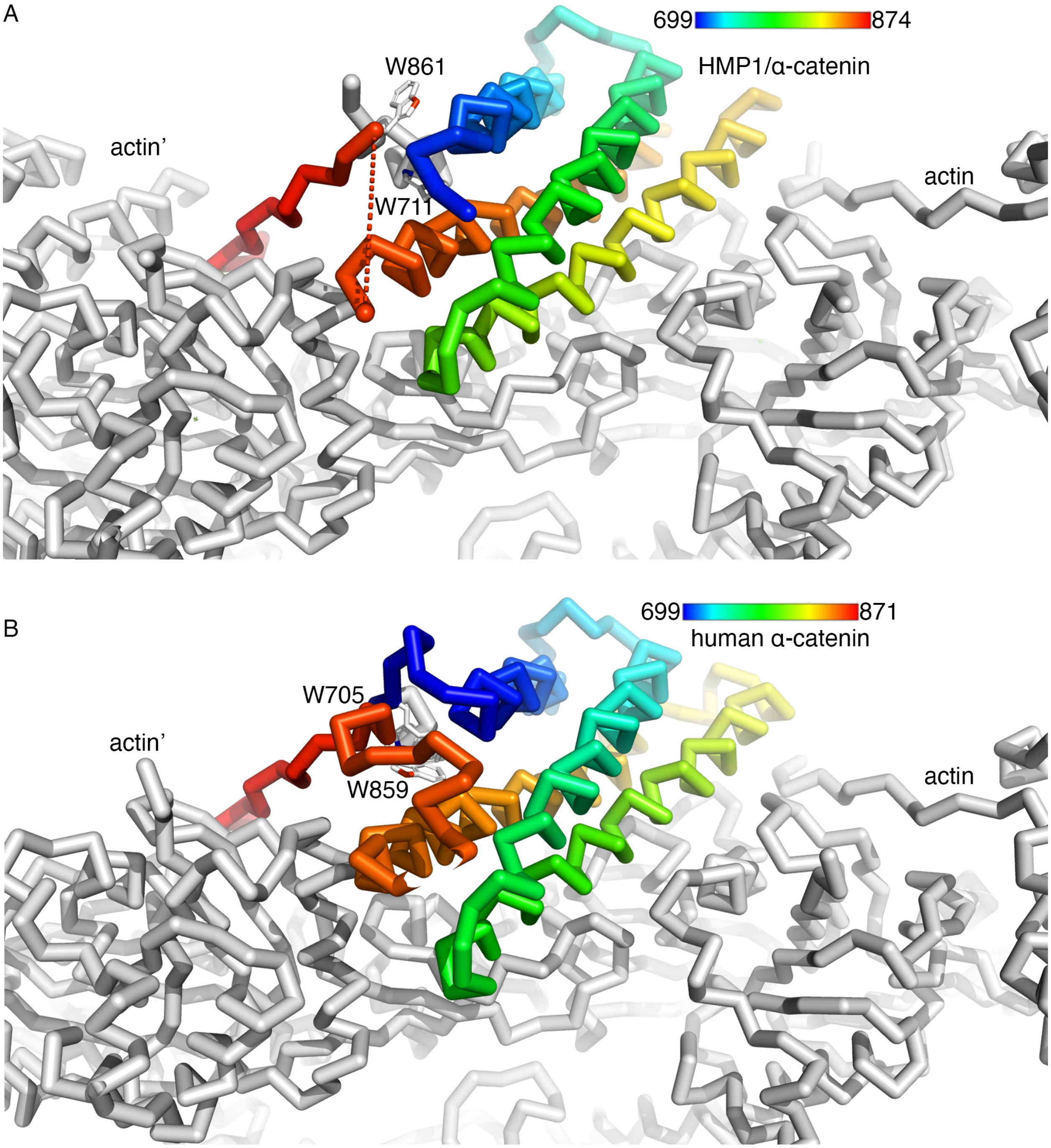

Supplementary Figure S3. The conformation of F-actin is similar when bound to roundworm or mammalian α-catenin

Superposition of the cryoEM structures of HMP1/α-catenin (magenta) bound to F-actin (colored spectrally) onto mammalian α-catenin bound to F-actin (PDB entry 6upv) (4) (gray). Neighboring actin subunits are labeled as actin and actin'. The Cα positions of the F-actin D-loop (residues 40-51) are shown as blue spheres in our F-actin bound to HMP1/α-catenin cryoEM structure.

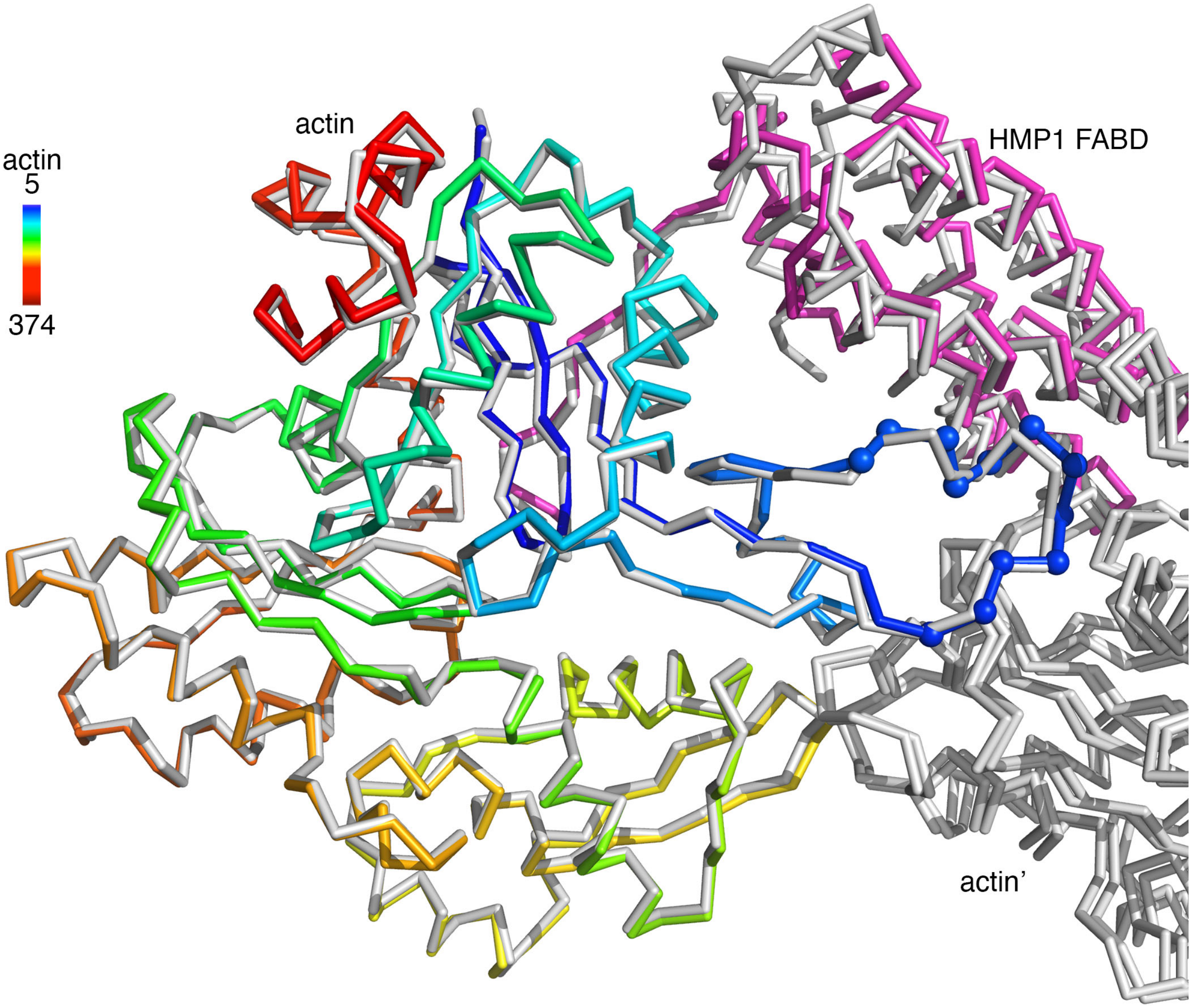

References

1. Altschul, S. F., Gish, W., Miller, W., Myers, E. W., and Lipman, D. J. (1990) Basic local alignment search tool. *J Mol Biol* **215**, 403-410

2. Shibahara, T., Hirano, Y., and Hakoshima, T. (2015) Structure of the free form of the N-terminal VH1 domain of monomeric α-catenin. *FEBS Lett* **589**, 1754-1760

3. Shao, X., Kang, H., Loveless, T., Lee, G. R., Seok, C., Weis, W. I., Choi, H. J., and Hardin, J. (2017) Cell-cell adhesion in metazoans relies on evolutionarily conserved features of the alpha-catenin.beta-catenin-binding interface. *J Biol Chem* **292**, 16477-16490

4. Mei, L., Espinosa de Los Reyes, S., Reynolds, M. J., Leicher, R., Liu, S., and Alushin, G. M. (2020) Molecular mechanism for direct actin force-sensing by α-catenin. *Elife* **9**
